# Comparative joint position error in patients with non-specific neck disorders and asymptomatic age-matched individuals

**DOI:** 10.4102/sajp.v75i1.568

**Published:** 2019-06-27

**Authors:** Jonathan Quartey, Markus Ernst, Ajediran Bello, Bertha Oppong-Yeboah, Emmanuel Bonney, Kow Acquaah, Felix Asomaning, Margaret Foli, Sandra Asante, Astrid Schaemann, Christoph Bauer

**Affiliations:** 1 Department of Physiotherapy, University of Ghana, Accra, Ghana; 2 Institute of Physiotherapy, Zurich University of Applied Sciences, Winterthur, Switzerland; 3 Physiotherapy Department, Korle-Bu Teaching Hospital, Accra, Ghana

**Keywords:** joint position error, proprioceptive, neck disability, age-matched, neck disability index, sex-matched

## Abstract

**Background:**

Neck pain is a common complaint worldwide and ranked seventh in 2010 as the cause of ‘years lived with disability’ in Ghana. Proprioceptive dysfunction, measured by joint position error (JPE) tests, indicates an association with neck pain frequency, dizziness and balance problems in patients.

**Objectives:**

To examine proprioceptive deficits of the neck using a laser pointer attached to the head.

**Methods:**

Twenty patients within the age group 21–60 years, with at least five points on the neck disability index (NDI), and 20 age- and sex-matched controls with less than five points on the NDI were recruited for this study. The JPE was determined wearing a headlight laser pointer directed towards a Cartesian coordinate system adjusted to *x*/*y* = 0/0, placed on a wall after returning from left and right rotation, flexion and extension. From starting in an upright sitting position, facing the Cartesian coordinate system, each participant performed five repetitions for each movement direction. The mean of five repetitions for each movement direction was calculated as absolute error (AE), constant error (CE) and variable error (VE).

**Results:**

Control participants showed larger JPE values for nearly all AE, CE and VE. After repositioning from flexion controls showed an approximately 0.6 ° larger median JPE, and the opposite for extension, with median differences between 1 ° and 2 °.

**Conclusion:**

The results of this study do not reveal any meaningful differences between patients with mild disabled neck movement compared with controls.

**Clinical implications:**

Joint position error testing does not seem useful for patients with mild neck disability.

## Introduction

Neck pain is a common complaint worldwide. Its point prevalence in sub-Saharan West Africa in 2010 was 4.1% in men and 6% in women and peaked around 45 years with 7% in men and 10% in women (Hoy et al. 2014). Its prevalence in southern Africa was about 1% more than the aforementioned values (Hoy et al. 2014). In a Nigerian population back pain and neck pain together were reported to add up to a prevalence of more than 16% and a third of this are from 60 or older (Gureje et al. 2007). In 2010 neck pain ranked seventh as the cause of ‘years lived with disability’ (YLD) in Ghana (http://vizhub.healthdata.org).

Proprioceptive dysfunction, typically measured by joint position error (JPE) tests, has been found to be associated with neck pain frequency (Lee et al. 2008), dizziness and balance problems in patients with whiplash injury (Treleaven 2011; Treleaven, Jull & Lowchoy 2006). Treleaven (2011) found JPE closely related to upper cervical spine complaints. Daenen et al. (2013) reported a predictive validity of proprioceptive dysfunction for long-term outcomes in patients who were moderately and severely affected by whiplash injury. Although recent reviews indicate that healthy controls differ in JPE measurements when compared with patients with whiplash injury, the differences in patients with idiopathic neck pain remain inconclusive (De Vries et al. 2015; De Zoete et al. 2017; Stanton et al. 2016).

Joint position error measurements are regarded to reflect proprioceptive functioning of the head and neck (Kristjansson & Treleaven 2009; Treleaven 2008, 2009). Afferent information derived by mechanoreceptors like muscle spindles, which are plentiful in the upper cervical spine, is directed to the central nervous system (Kristjansson & Treleaven 2009; Treleaven 2008, 2009) and via reflex connections to the visual, the vestibular and somatosensory systems. Thus, eyes, head and body movements are guided and constantly rearranged to modify the input system of the neuromuscular pathway. A mismatch of information derived by these systems can lead to symptoms like dizziness or unsteadiness, which are especially prevalent in patients with whiplash injury (Treleaven 2011). Whether a disturbed JPE sense due to injury or mechanical disturbances of the neck is the reason for these symptoms should be evaluated clinically by examination of all contributing systems (Kristjansson & Treleaven 2009; Treleaven 2008, 2009, 2011).

Joint position error has been examined since the early nineties, when Revel, Andre-Deshays and Minguet (1991) mentioned a best specific and sensitive cut-off of 4.5 ° between participants with neck pain and healthy controls in horizontal and sagittal plane movements. Values of more than four and a half degrees (> 4.5 °) were more frequently found in patients with neck pain (Revel et al. 1991). This cut-off value has been reported to be relatively robust in some studies (De Hertogh et al. 2008; Heikkila & Astrom 1996; Humphreys & Irgens 2002; Kristjansson et al. 2001; Pinsault et al. 2008; Rix & Bagust 2001; Roren et al. 2008); however, a recent meta-analysis by De Zoete et al. (2017) reported a large variability of values between patients with idiopathic neck pain and healthy controls across studies, which provide indications that a 4.5 ° threshold might be specific to detect healthy or asymptomatic participants but does not mean that it is sensitive enough. It also indicates that probably different cut-offs for flexion or extension and rotation exist. Another meta-analysis by Stanton et al. in 2016 reported a moderate overall standardised mean difference of 0.44 between patients with idiopathic pain and healthy controls. Both reviews (De Zoete et al. 2017; Stanton et al. 2016) reported a possible expectation bias in most studies since investigators were most often not adequately blinded to patient condition. With no known risk previously reported, JPE can easily be examined in daily clinical practice, by using instruments such as laser pointers attached to an Alice band or a helmet (Clark, Roijezon & Treleaven 2015; Roijezon, Clark & Treleaven 2015).

Another review on the effectiveness of proprioceptive exercising for improving sensorimotor control for several health conditions by Aman et al. (2014) demonstrated general benefits of ‘proprioceptive training’, but it included only one study with a neck pain condition. A more recent randomised controlled trial by Treleaven et al. (2016) reported on the effectiveness of neck-specific exercising in patients with chronic whiplash injury on proprioceptive and disability outcomes. Patients and therapists need assessment instruments to reliably determine values of proprioceptive functioning. The assessment should also be able to demonstrate differences between known groups, which are supposed to differ for the trait (proprioception) measured. It appears that there were no similar studies that have examined proprioceptive dysfunction by using JPE in Ghanaians or even African populations suffering from neck pain. This study may therefore form the basis to enhance the possibilities of similar studies with useful variations.

The aim of this study was to examine the effect of neck pain and disability on JPE of the cervical spine in a Ghanaian population.

## Methods

This cross-sectional study involving 40 participants (20 patients with neck pain and 20 participants without neck pain) was conducted at the outpatient unit, physiotherapy Department, Korle-Bu Teaching hospital, Accra, Ghana. The unit sees patients with a wide variety of conditions including cervical spondylosis on an outpatient basis.

Patients with neck pain who reported to the outpatient physiotherapy unit and were within the age group of 21–60 years, suffering from sub-acute (≥ 6 weeks) or chronic (> 3 months) non-specific head and neck pain with disability due to neck pain (with at least five points on the neck disability index [NDI]), were recruited for the study as cases. Gender- and age-matched participants (within ± 3 years of age) who had no neck pain, no relevant history of neck or upper limb pain or injury over the last 3 years that limited their function or required treatment from a health professional with a score of less than five points on the NDI were recruited as controls.

**FIGURE 1:**
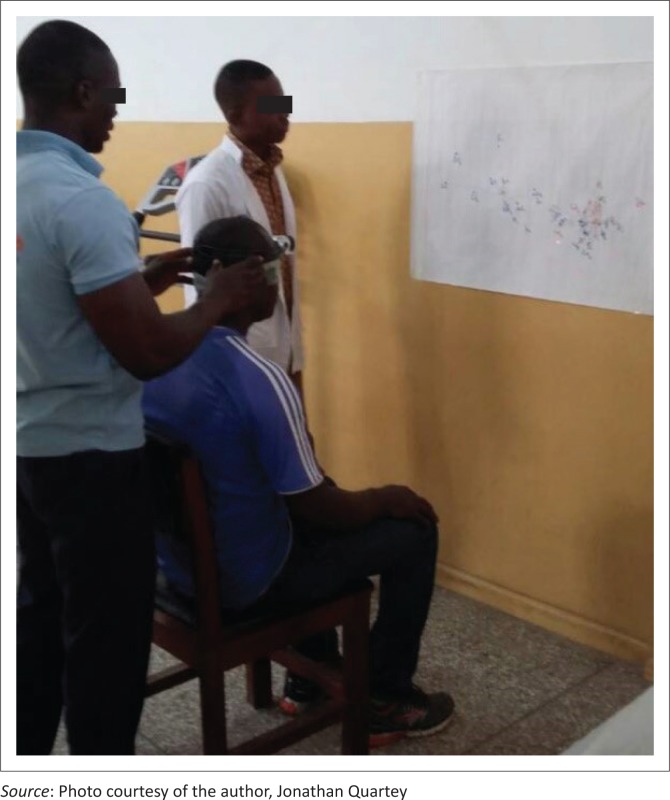
Participant seated wearing a headlight laser pointer on the head.

Participants were requested to sit upright, in a neutral but comfortable head position (NHP) with hips and knees flexed at 90 ° at a fixed distance of 90 cm to a target, wearing a headlight laser pointer on the head (Figure 1). The target, a white piece of paper with a starting point (reticle), in the middle (Figure 1) was placed on a wall and adjusted according to the upright position of the participant, by using adhesive tape.

Participants were blindfolded and asked to move their head and neck from NHP into approximately 50% of maximal range in one of four directions, which were left and right rotations (LR and RR), flexion (F) and extension (E). Participants were asked to move slowly, in order not to stimulate the vestibular organ.

Participants were then asked to actively and slowly reposition to the NHP while still blindfolded and verbally asked to indicate when they perceived that NHP had been attained.

One of two authors indicated the end position of all movements (F, E, RL, RR) at the target by using a pen. One of the other authors helped the blindfolded participants after each repetition to find the accurate starting position again. After repetition the position was readjusted actively by ensuring that the laser point rested at the starting point (0 ° / 0 °) again. An assessor, observing the participant, assured upright position. Five repetitions for each direction (F, E, RL and RR) were carried out by each participant as recommended in previous studies (Demaille-Wlodyka et al. 2007; Pinsault et al. 2008; Strimpakos et al. 2006). Short relaxations/distractions were allowed after each repetition.

After five repetitions the authors removed the target and measured the deviations from the target position for each repetition on both axes (ordinate and abscissae) by using a common ruler with millimetre distances. The whole procedure including short breaks took 10–15 min to complete (per participant).

**FIGURE 2:**
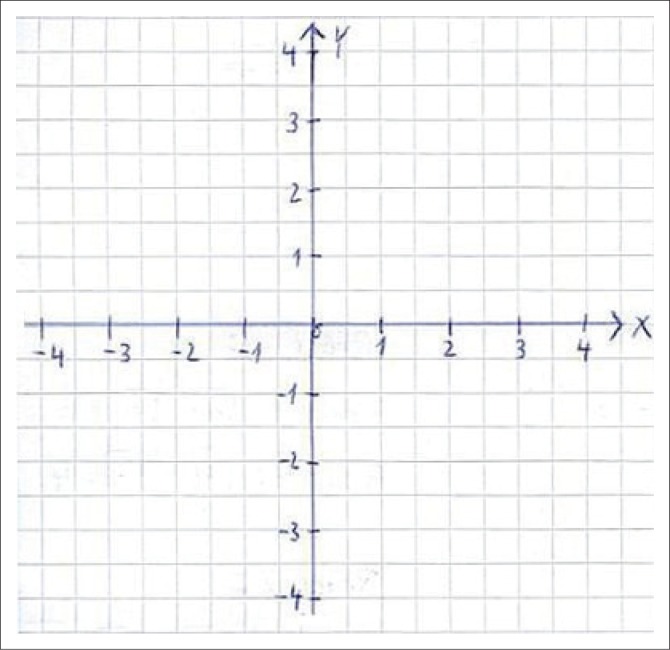
Cartesian coordinate system.

Values for *x* (abscissa) and *y* (ordinate) were written and listed with a minus sign as a prefix, if indicated and according to the Cartesian coordinate system (Figure 2). The resultant (*d*) of *x* and *y* values were then calculated by using the Pythagoras theorem. Centimetre values for the resultant (*d*) were then converted to degrees with the formula (Chen & Treleaven 2013; Roren et al. 2009):

$$\Theta =\tan^{-1}(\text{error distance}/90\text{ cm})$$

Absolute (AE), constant (CE) and variable (VE) errors during F, E, RR and RL were calculated and reported as degrees. Absolute error represents the mean in error magnitude of five repetitions irrespective of the direction of error in terms of undershooting (negative values) or overshooting (positive values), CE represents the mean in error magnitude of five repetitions incorporated in the direction of error and VE represents the variability of subjects’ performance (Hill et al. 2009).

The generalisability theory (Brennan 2001) with the design *p × t* (*participants *×* trials*) was used as a framework to estimate reliability of trunk movement measures, based on the linear model:

$$X_{pt}=\mu +v_{p}+v_{t}+v_{pt}$$

with *μ* representing the global mean and *v* any one of the three components.

The index of dependability Φ was calculated:

$$\Phi =\frac{\sigma_{p}^{2}}{\sigma_{p}^{2}+\frac{\sigma_{t}^{2}}{n_{t}}+\frac{\sigma_{pt}^{2}}{n_{t}}}$$

with σ being the variance and *n* the number of the corresponding components. The index of dependability (Φ) was interpreted as: < 0.25 – little, 0.26–0.49 – low, 0.50–0.69 – moderate, 0.70–0.89 – high and > 0.90 – very high reliability (Carter, Lubinsky & Domholdt 2005). An index of dependability (Φ) ≥ 0.70 was interpreted as sufficient. D-studies (Brennan 2001) were simulated where the number of trials varied up to 10 trials and number of days varied across 2 days, which represent acceptable measurement strategies. Thereby, the number of required trials per day to achieve high reliability was evaluated.

The coefficient of variation (CV) (Hopkins 2000) was also calculated:

$$CV=\frac{\sigma_{diff}}{\sqrt{n_{d}}*\bar{x}}*100$$

with $\bar{x}$ being the grand mean and *σ*_*diff*_ being the standard deviation of the differences between days and calculated from the mean of seven trials per day. The CV values were rated as follows: > 10% not reliable, 6% – 10% adequately reliable and 5% highly reliable. Coefficient of variations ≤ 10% were construed as sufficient (Suni, Rinne & Ruiz 2014).

The diagnostic value of a variable was assessed by the index of dependability (Φ), whereas the ability to detect changes over time was evaluated by the CV. Data from both populations were pooled for this analysis. Wilcoxon tests between cases and matched controls for the resultant (*d*) for all dependent variables (AE, CE; VE) were performed. All analyses were performed by using the R statistical software, version 3.2.3.

### Ethical considerations

Ethical approval for this study was sought and obtained (SBAHS – ET./AA/2014–2015) from the Ethics and Protocol Review Committee of the School of Biomedical and Allied Health Sciences, University of Ghana. Permission was also sought and obtained from the Physiotherapy Department of Korle-Bu Teaching Hospital. Written informed consent was obtained from participants before measurements were carried out.

## Results

Forty participants (20 cases and 20 controls) were recruited and measured. Each group consisted of 15 women and five men. Baseline characteristics are shown in Table 1.

**TABLE 1:** Baseline characteristics of participants.

Variable	Cases	Controls	*p*
Total number (female participants)	20 (15)	20 (15)	1.00
Age in years (SD)	51.70 (9.90)	52.1 (8.30)	0.90
Height in cm (SD)	165.00 (5.40)	165.0 (7.60)	0.98
Weight in kg (SD)	73.50 (8.03)	79.1 (18.20)	0.28
NDI in points (SD)	6.45 (0.89)	2.4 (1.00)	4.569e-16
Duration of complaints in weeks (SD)	18.50 (17.50)	na	na

Note: All values are mean values and standard deviations (SD), otherwise indicated. *p*-values are derived from unpaired *t*-tests and chi square tests (sex distribution).

NDI, Neck Disability Index; na, not applicable.

The controls showed larger JPE values for the resultant (vector of *x* and *y* values) for nearly any AE, CE and VE as well as movement direction, apart from the RR CE and flexion VE. Differences were small for LR and RR JPEs. For flexion VE, cases had an approximately 0.6 ° larger median JPE, and the extension CE and AE showed median differences close to 1 ° and 2 ° respectively, which revealed a statistical significance (Table 2).

**TABLE 2:** Median differences of scores.

Direction (error)	Values	Patients (*n* = 20)	Controls (*n* = 20)	*p* (Wilcoxon-test)
LR (AE)	Median (IQR)	4.41 (2.59)	4.59 (2.56)	0.24
LR (CE)	Median (IQR)	2.55 (2.97)	3.46 (2.54)	0.25
LR (VE)	Median (IQR)	1.79 (1.08)	1.65 (0.81)	0.70
RR (AE)	Median (IQR)	4.28 (3.71)	4.11 (3.11)	0.88
RR (CE)	Median (IQR)	2.38 (3.73)	2.60 (2.33)	0.74
RR (VE)	Median (IQR)	1.57 (0.83)	1.65 (1.43)	0.99
F(AE)	Median (IQR)	4.99 (1.89)	5.13 (5.46)	0.55
F (CE)	Median (IQR)	3.30 (2.23)	3.82 (3.91)	0.86
F (VE)	Median (IQR)	1.65 (0.93)	1.06 (1.02)	0.13
E (AE)	Median (IQR)	3.38 (1.76)	5.09 (2.67)	0.02*
E (CE)	Median (IQR)	1.81 (1.96)	2.86 (2.92)	0.17
E (CE)	Median (IQR)	1.42 (0.59)	1.57 (1.26)	0.55

AE, absolute error; CE, constant error; VE, variable error; LR, left rotation; RR, right rotation; F, flexion; E, extension; IQR, interquartile range.

* , Statistically significant

Table 3 shows the summary of the grand mean, the index of dependability (Φ) coefficients, the number of trials averaged on one measurement day, which are needed to gain Φ ≥ 0.70 and the CV for each variable. On average, two trials on one day were sufficient to reach high reliability, indicating the diagnostic suitability of those variables should differences between groups exist. The CVs exceeded 10% of the grand mean indicating that these variables are not suitable to measure changes over time.

**TABLE 3:** Reliability of a single measure, number of trials averaged on one day, needed to achieve high reliability and coefficient of variation.

Test	Mean (SEM)	Φ one trial	Number of trials Φ > 0.7 One day	CV (%)
Flexion	5.82 (4.01)	0.77	1	69
Extension	4.81 (3.82)	0.60	2	79
LR	5.14 (3.98)	0.62	2	77
RR	4.98 (3.93)	0.61	2	78

Φ, index of dependability; CV, coefficient of variation, SEM, standard error of the measurement.

## Discussion

Our study showed minor differences in JPE measurements of the neck in a Ghanaian population of patients with neck pain with mild disability compared with age- and sex-matched asymptomatic controls.

However, the asymptomatic controls showed larger values for most of the AE, CE and VE, although differences were small, except for extension (AE), which was of no statistical significance.

This could be the first study that has measured JPE of the neck in a Ghanaian or probably African population. Most previous studies were conducted in North America, Western Europe or Australia.

The AEs of patients with neck pain showed comparable values to pooled mean values reported by a recent meta-analysis (De Zoete et al. 2017). With values of 4.0 ° – 4.5 ° for repositioning after rotation and 3.4 ° after extension, the outcome values of our study are within reported values by De Zoete et al. (2017).

However, values for flexion and extension for the asymptomatic participants and flexion values from patients with neck pain exceeded those reported in that meta-analysis (De Zoete et al. 2017).

Our study is not the only one that has reported larger errors for the control group. Rix and Bagust (2001) reported similar values for LR and RR and larger values for extension in controls. In contrast to the results of this study Rix and Bagust (2001) reported statistically significant differences for *F*.

The study by Rix and Bagust (2001) consisted of 11 participants in each group, with an almost equal distribution of men and women, but with participants who were on average 10 years younger than those of this study. They did not use the NDI, but reported pain intensity values for the patient group of 5.1 ± 1.9 on an 11-point numeric rating scale on the day of measurement. For JPE measurement they used a helmet with the laser pointer attached to its top; participants had to move into full range of motion for all directions measured.

Rix and Bagust (2001) reported that this full range of motion movement and the speed of their repositioning movement could have been possible factors for not finding meaningful results.

Zito, Jull and Story (2006) reported no differences between patients with migraine, participants suffering from cervicogenic headache and controls. Most mean values reported by these authors exceeded the 4.5° cut-off, but for all three groups and like in this study the control participants showed larger values although not statistically significant. Zito et al. (2006) reported that neck pain from traumatic conditions might have led to group differences that are more distinct. However, this assumption could not be determined in any of those studies comparing patients with whiplash injury to controls, as De Vries et al. (2015) reported in a systematic review. Sterling et al. (2003) demonstrated differences, but only for patients suffering from whiplash injury with severe pain and disability, indicated by > 30 points on the NDI, and only for RR. However, Sterling et al. (2003) did not report differences between patients with mild or moderate disability and control participants.

More recent studies (Chen & Treleaven 2013; Elsig et al. 2014; Meisingset et al. 2015) have also shown a large overlap between JPE values from patients with neck pain and controls, and even in patients reporting larger pain and disability values than the participants of this study. Even the presence of subjectively perceived dizziness as shown by Chen and Treleaven (2013) could not demonstrate larger differences for the classic JPE test to NHP.

De Zoete et al. (2017) pooled data from 22 primarily cross-sectional case control studies with 340 patients with idiopathic neck pain and 630 healthy controls and reported a median group difference of approximately 0.5 °. This clearly puts the measurement or the population measured into question.

Studies that examined the responsiveness of JPE tests in a clinical population were not found. A minimal detectable change for returning to NHP from extension was examined by Alahmari et al. (2017) for a mixed group of patients with and without neck pain by using a similar protocol used in this study.

Alahmari et al. (2017) reported standard error of measurements (SEMs) of approximately 2 ° and minimal detectable changes of approximately 5 °. A larger homogeneity compared with the study carried out by Alahmari et al. as expressed in the NDI score and the smaller sample size (*n* = 69 for Alahmari et al. and *n* = 40 for this study) might have led to larger SEMs of approximately 4 ° within our study as shown in Table 3 (Alahmari et al. 2017; Streiner & Norman 2008).

The high Φ coefficients indicate diagnostic ability of the variables of this study and compare favourably to other protocols (Juul et al. 2013; Lee et al. 2006; Pinsault et al. 2008; Strimpakos et al. 2006; Wibault et al. 2013), whereas the high CVs indicate that the protocol of our study might not be suitable to monitor changes over time. Improving the standardisation of the protocol of this study regarding the placement of the laser pointer, target or sitting position may reduce the CV in future studies.

Cases had to fulfil inclusion criteria of at least five points on the NDI (Vernon & Mior 1991). Five points has been promoted as cut-off to differentiate mild neck pain and disability from no pain and disability as indicated by Vernon an Mior (1991). However, controls were described as eligible with a value of 10 points or 20% in a JPE study by Wibault et al. (2013). Within our study, the mean NDI values for cases and controls were 2.4 and 6.5 points, respectively (Table 1), and all the cases in this study would have fulfilled the eligibility criteria to be a ‘control’ in the study by Wibault et al. (2013). Besides this, the relationship of the NDI and JPE does not seem to have been examined by any study so far. So it might be questionable whether the NDI or the cut-off of 5 points is suitable to determine meaningful JPE differences in participants with NDI scores ranging 0–5 compared with 5–10 points

The duration of symptoms might be a better indicator to find differences between patients with neck pain and controls. Cheng et al. (2010) reported on average 50% larger values for flexion and twice the values for extension in young patients suffering on average for 4 years from non-traumatic neck pain (Cheng et al. 2010). An unknown history of neck pain in the control group might have also influenced results, as shown in a similar case–control study by Teng et al. (2007). Those with a history of, but no current neck pain, had similar JPEs as participants in the neck pain sample (Teng et al. 2007).

### Limitations

As the investigators were not blinded to the participants’ condition, this might have led to an expectation bias as mentioned by the aforementioned reviews of Stanton et al. (2016) and De Zoete et al. (2017). The testing method used required accurate notation of each reposition manoeuvre on the paper and subsequently measured the distance on *x*- and *y*-axis. Inaccuracies during these recordings might have occurred but are expected to be equally distributed between groups, although measurements and data entry were double-checked.

## Conclusion

This study showed no differences between patients with neck pain with mild disability and asymptomatic control participants in a Ghanaian population for cervical joint reposition error testing after rotation and flexion or extension movements. Thus, this kind of measurement cannot be recommended for clinical situations with similar neck pain patients.

## References

[CIT0001] Alahmari K., Reddy R.S., Silvian P., Ahmad I., Nagaraj V. & Mahtab M., 2017, ‘Intra- and inter-rater reliability of neutral head position and target head position tests in patients with and without neck pain’, *Brazilian Journal of Physical Therapy* 21, 259–267. 10.1016/j.bjpt.2017.05.003 28558952 PMC5537472

[CIT0002] Aman J. E., Elangovan N., Yeh I. L. & Konczak J., 2014, ‘The effectiveness of proprioceptive training for improving motor function: A systematic review’, *Frontiers in Human Neuroscience* 8, 1075. 10.3389/fnhum.2014.01075 PMC4309156 25674059

[CIT0003] Brennan R.L., 2001, *Generalizability theory*, Springer-Verlag, Berlin.

[CIT0004] Carter R.E., Lubinsky J. & Domholdt E., 2005, *Rehabilitation research: Principles and applications*, Elsevier Saunders, St. Louis, MO.

[CIT0005] Chen X. & Treleaven J., 2013, ‘The effect of neck torsion on joint position error in subjects with chronic neck pain’, *Manual Therapy* 18, 562–567. 10.1016/j.math.2013.05.015 23810427

[CIT0006] Cheng C.H., Wang J.L., Lin J.J., Wang S.F. & Lin K.H., 2010, ‘Position accuracy and electromyographic responses during head reposition in young adults with chronic neck pain’, *Journal of Electromyography & Kinesiology* 20, 1014–1020. 10.1016/j.jelekin.2009.11.002 20005126

[CIT0007] Clark N.C., Roijezon U. & Treleaven J., 2015, ‘Proprioception in musculoskeletal rehabilitation. Part 2: Clinical assessment and intervention’, *Manual Therapy* 20, 378–387. 10.1016/j.math.2015.01.009 25787919

[CIT0008] Daenen L., Nijs J., Raadsen B., Roussel N., Cras P. & Dankaerts W., 2013, ‘Cervical motor dysfunction and its predictive value for long-term recovery in patients with acute whiplash-associated disorders: A systematic review’, *Journal of Rehabilitation Medicine* 45, 113–122. 23307298 10.2340/16501977-1091

[CIT0009] De Hertogh W., Vaes P., Beckwee D., Van Suijlekom H., Duquet W. & Van Roy P., 2008, ‘Lack of impairment of kinaesthetic sensibility in cervicogenic headache patients’, *Cephalalgia* 28, 323–328. 18284421 10.1111/j.1468-2982.2007.01505.x

[CIT0010] Demaille-Wlodyka S., Chiquet C., Lavaste J.F., Skalli W., Revel M. & Poiraudeau S., 2007, ‘Cervical range of motion and cephalic kinesthesis: Ultrasonographic analysis by age and sex’, *Spine (Phila Pa 1976)* 32, E254–E261. 10.1097/01.brs.0000259919.82461.57 17426621

[CIT0011] De Vries J., Ischebeck B.K., Voogt L.P., Van Der Geest J.N., Janssen M., Frens M.A. et al., 2015, ‘Joint position sense error in people with neck pain: A systematic review’, *Manual Therapy* 20, 736–744. 10.1016/j.math.2015.04.015 25983238

[CIT0012] De Zoete R.M.J., Osmotherly P.G., Rivett D.A., Farrell S.F. & Snodgrass S.J., 2017, ‘Sensorimotor control in individuals with idiopathic neck pain and healthy individuals: A systematic review and meta-analysis’, *Archives of Physical Medicine and Rehabilitation* 98, 1257–1271. 10.1016/j.apmr.2016.09.121 27771360

[CIT0013] Elsig S., Luomajoki H., Sattelmayer M., Taeymans J., Tal-Akabi A. & Hilfiker R., 2014, ‘Sensorimotor tests, such as movement control and laterality judgment accuracy, in persons with recurrent neck pain and controls. A case-control study’, *Manual Therapy* 19, 555–561. 10.1016/j.math.2014.05.014 24957711

[CIT0014] Gureje O., Akinpelu A.O., Uwakwe R., Udofia O. & Wakil A., 2007, ‘Comorbidity and impact of chronic spinal pain in Nigeria’, *Spine (Phila Pa 1976)* 32, E495–E500. 10.1097/BRS.0b013e31810768fc 17762283

[CIT0015] Heikkila H. & Astrom P.G., 1996, ‘Cervicocephalic kinesthetic sensibility in patients with whiplash injury’, *Scandinavian Journal of Rehabilitation Medicine* 28, 133–138. 8885035

[CIT0016] Hill R., Jensen P., Baardsen T., Kulvik K., Jull G. & Treleaven J., 2009, ‘Head repositioning accuracy to neutral: A comparative study of error calculation’, *Manual Therapy* 14, 110–114. 10.1016/j.math.2008.02.008 18502679

[CIT0017] Hopkins W.G., 2000, ‘Measures of reliability in sports medicine and science’, *Sports Medicine* 30, 1–15. 10.2165/00007256-200030010-00001 10907753

[CIT0018] Hoy D., March L., Woolf A., Blyth F., Brooks P., Smith E. et al., 2014, ‘The global burden of neck pain: Estimates from the global burden of disease 2010 study’, *Annals of the Rheumatic Diseases* 73, 1309–1315. 10.1136/annrheumdis-2013-204431 24482302

[CIT0019] Humphreys B.K. & Irgens P.M., 2002, ‘The effect of a rehabilitation exercise program on head repositioning accuracy and reported levels of pain in chronic neck pain subjects’, *Journal of Whiplash & Related Disorders* 1, 99–112.

[CIT0020] Juul T., Langberg H., Enoch F. & Sogaard K., 2013, ‘The intra- and inter-rater reliability of five clinical muscle performance tests in patients with and without neck pain’, *BMC Musculoskeletal Disorders* 14, 339 10.1186/1471-2474-14-339 24299621 PMC4219589

[CIT0021] Kristjansson E., Dall’Alba P. & Jull G., 2001, ‘Cervicocephalic kinaesthesia: Reliability of a new test approach’, *Physiother apy Research International* 6, 224–235. 10.1002/pri.230 11833244

[CIT0022] Kristjansson E. & Treleaven J., 2009, ‘Sensorimotor function and dizziness in neck pain: Implications for assessment and management’, *Journal of Orthopaedic & Sports Physical Therapy* 39, 364–377. 10.2519/jospt.2009.2834 19411769

[CIT0023] Lee H., Teng C., Chai H. & Wang S., 2006, ‘Test-retest reliability of cervicocephalic kinesthetic sensibility in three cardinal planes’, *Manual Therapy* 11, 61–68. 10.1016/j.math.2005.03.008 15922647

[CIT0024] Lee H., Wang J., Yao G. & Wang S., 2008, ‘Association between cervicocephalic kinesthetic sensibility and frequency of subclinical neck pain’, *Manual Therapy* 13, 419–425. 10.1016/j.math.2007.04.001 17544825

[CIT0025] Meisingset I., Woodhouse A., Stensdotter A.-K., Stavdahl Ø., Lorås H., Gismervik S. et al., 2015, ‘Evidence for a general stiffening motor control pattern in neck pain: A cross sectional study’, *BMC Musculoskeletal Disorders* 16, 56 10.1186/s12891-015-0517-2 25888215 PMC4377005

[CIT0026] Pinsault N., Fleury A., Virone G., Bouvier B., Vaillant J. & Vuillerme N., 2008, ‘Test-retest reliability of cervicocephalic relocation test to neutral head position’, *Physiotherapy Theory and Practice* 24, 380–391. 10.1080/09593980701884824 18821444

[CIT0027] Revel M., Andre-Deshays C. & Minguet M., 1991, ‘Cervicocephalic kinesthetic sensibility in patients with cervical pain’, *Archives of Physical Medicine and Rehabilitation* 72, 288–291. 2009044

[CIT0028] Rix G.D. & Bagust J., 2001, ‘Cervicocephalic kinesthetic sensibility in patients with chronic, nontraumatic cervical spine pain’, *Archives of Physical Medicine and Rehabilitation* 82, 911–919. 10.1053/apmr.2001.23300 11441377

[CIT0029] Roijezon U., Clark N.C. & Treleaven J., 2015, ‘Proprioception in musculoskeletal rehabilitation. Part 1: Basic science and principles of assessment and clinical interventions’, *Manual Therapy* 20, 368–377. 10.1016/j.math.2015.01.008 25703454

[CIT0030] Roren A., Mayoux-Benhamou M.A., Fayad F., Poiraudeau S., Lantz D. & Revel M., 2009, ‘Comparison of visual and ultrasound based techniques to measure head repositioning in healthy and neck-pain subjects’, *Manual Therapy* 14, 270–277. 10.1016/j.math.2008.03.002 18514016

[CIT0031] Stanton T.R., Leake H.B., Chalmers K.J. & Moseley G.L., 2016, ‘Evidence of impaired proprioception in chronic, idiopathic neck pain: Systematic review and meta-analysis’, *Physical Therapy* 96, 876–887. 10.2522/ptj.20150241 26472296 PMC4897597

[CIT0032] Sterling M., Jull G., Vicenzino B., Kenardy J. & Darnell R., 2003, ‘Development of motor system dysfunction following whiplash injury’, *Pain* 103, 65–73. 10.1016/S0304-3959(02)00420-7 12749960

[CIT0033] Streiner D.L. & Norman G.R., 2008, *Health measurement scales*, Oxford University Press, Oxford, UK.

[CIT0034] Strimpakos N., Sakellari V., Gioftsos G., Kapreli E. & Oldham J., 2006, ‘Cervical joint position sense: An intra- and inter-examiner reliability study’, *Gait Posture* 23, 22–31. 10.1016/j.gaitpost.2004.11.019 16311191

[CIT0035] Suni J., Rinne M. & Ruiz J., 2014, ‘Retest repeatability of motor and musculoskeletal fitness tests for public health monitoring of adult populations’, *Journal of Novel Physiotherapies* 4, 194.

[CIT0036] Teng C.C., Chai H., Lai D.M. & Wang S.F., 2007, ‘Cervicocephalic kinesthetic sensibility in young and middle-aged adults with or without a history of mild neck pain’, *Manual Therapy* 12, 22–28. 10.1016/j.math.2006.02.003 16777468

[CIT0037] Treleaven J., 2008, ‘Sensorimotor disturbances in neck disorders affecting postural stability, head and eye movement control’, *Manual Therapy* 13, 2–11. 10.1016/j.math.2007.06.003 17702636

[CIT0038] Treleaven J., 2009, ‘Sensorimotor disturbances in neck disorders affecting postural stability, head and eye movement control’, *New Zealand Journal of Physiotherapy* 37, 152–152.

[CIT0039] Treleaven J., 2011, ‘Dizziness, unsteadiness, visual disturbances, and postural control: Implications for the transition to chronic symptoms after a whiplash trauma’, *Spine (Phila Pa 1976)* 36, S211–S217. 10.1097/BRS.0b013e3182387f78 22020615

[CIT0040] Treleaven J., Jull G. & Lowchoy N., 2006, ‘The relationship of cervical joint position error to balance and eye movement disturbances in persistent whiplash’, *Manual Therapy* 11, 99–106. 10.1016/j.math.2005.04.003 15919229

[CIT0041] Treleaven J., Peterson G., Ludvigsson M.L., Kammerlind A.-S. & Peolsson A., 2016, ‘Balance, dizziness and proprioception in patients with chronic whiplash associated disorders complaining of dizziness: A prospective randomized study comparing three exercise programs’, *Manual Therapy* 22, 122–130. 10.1016/j.math.2015.10.017 26678652

[CIT0042] Vernon H. & Mior S., 1991, ‘The Neck Disability Index: A study of reliability and validity’, *Journal of Manipulative & Physiological Therapeutics* 14, 409–415. 10.1037/t35122-000 1834753

[CIT0043] Wibault J., Vaillant J., Vuillerme N., Dedering A. & Peolsson A., 2013, ‘Using the cervical range of motion (CROM) device to assess head repositioning accuracy in individuals with cervical radiculopathy in comparison to neck- healthy individuals’, *Manual Therapy* 18, 403–409. 10.1016/j.math.2013.02.004 23473752

[CIT0044] Zito G., Jull G. & Story I., 2006, ‘Clinical tests of musculoskeletal dysfunction in the diagnosis of cervicogenic headache’, *Manual Therapy* 11, 118–129. 10.1016/j.math.2005.04.007 16027027

